# Continuous cardiac monitoring in epilepsy: an implantable loop manual activation algorithm for improving ECG signal acquisition accuracy

**DOI:** 10.1186/s12872-024-03721-5

**Published:** 2024-01-13

**Authors:** Karapet Davtyan, Svetlana Serdyuk, Arpi Topchyan, Georgiy Simonyan, Maria Kharlap, Sergey Burd

**Affiliations:** 1 National Medical Research Center for Therapy and Preventive Medicine, Petroverigskiy Lane 10-3, Moscow, 101990 Russia; 2 Pirogov Russian Research Medical University, 117997, Ostrovitianov str. 1, Moscow, Russia

**Keywords:** Cardiac arrhythmias, Continuous monitoring, Seizure, False-positive arrhythmias

## Abstract

**Background:**

The muscle artifacts, caused by prominent muscle contractions, mimicking cardiac arrhythmias, might compromise the ECG signal quality and the implantable loop recorder memory capacity in patients with epilepsy. We developed an epileptic seizures clinical pattern-based implantable loop recorder manual activation algorithm, presenting its real-world efficacy here.

**Methods:**

One hundred ninety-three patients (18–60 years) with drug-resistant focal epilepsy were consecutively enrolled and underwent a subcutaneous loop recorder implantation. Patients with focal onset-aware seizures and patients with focal impaired awareness seizures /bilateral tonic-clonic seizures without aura were recommended to use the activator once - just after the episode. Patients with focal impaired awareness seizures/bilateral tonic-clonic seizures with aura, the caregivers of patients experiencing status epilepticus, were advised to use the activator twice - during the aura and after the episode/ regaining consciousness.

**Results:**

Six thousand four hundred ninety-four ECG traces (4826 - auto-triggered events, 1668 - person-activated events) were recorded and analyzed. The rate of true positive events in the person-activated group was statistically higher than in the autoactivation group (72.5% vs.19.4%, *p* < 0.0001). Person-activated false-positive events were observed in 30.5% of patients with focal impaired awareness seizures and 27.7% in patients with bilateral tonic-clonic seizures. The highest rate of false-positive events (61.5%) was detected in patients undergoing epileptic status, and the lowest rate (3.8%) - was in patients with focal onset aware seizures. The rate of false-positive events was significantly higher in patients with impaired awareness seizures without aura both in focal impaired awareness (45.5% vs. 19.3%, *p* < 0.0001) and bilateral tonic-clonic seizure groups (38.8% vs. 5.9%, *p* < 0.0001).

**Conclusions:**

Arrhythmias with varying clinical outcomes are expected in epilepsy patients and have been monitored continuously. The specified loop recorder external activation algorithm can improve the clinically relevant cardiac arrhythmia detection accuracy in epilepsy patients and the value of future studies.

## Introduction

Despite the observed decrease in the epilepsy burden during the last years, epilepsy continues to be associated with high morbidity, mortality, and disability rates [1]. Arrhythmias with varying clinical outcomes are expected in epilepsy patients [2]. In 2015, we initiated a prospective study to evaluate the frequency and type of cardiac arrhythmias via long-term continuous cardiac monitoring in a large sample of patients (*n* = 193) with drug-resistant epilepsy [3]. It is well-known that muscle artifacts, caused by prominent muscle contractions, are common during epileptic seizures and might compromise the ECG signal quality [4, 5], mimicking cardiac arrhythmias. This limitation of detecting pseudo-arrhythmia would compromise the implantable loop recorder (ILR) memory capacity. Foreseeing this critical limitation, our team of experienced cardiologists, cardiac electrophysiologists, and neurologists developed an ILR manual activation algorithm based on an analysis of the clinical pattern of epileptic seizures. Our algorithm is the first systemized approach to overcome this limitation. Moreover, it was tested in a large sample of patients, and here we present a detailed analysis of our algorithm’s real-world efficacy. We believe it can help improve the clinically relevant cardiac arrhythmia detection accuracy and the value of future studies on this subject.

## Materials and methods

The study design was detailly described previously [3] Briefly, 193 patients (age 18–60 years) with video-EEG monitoring confirmed drug-resistant focal epilepsy, having at least one seizure per month, were consecutively enrolled in this prospective observative study and underwent a subcutaneous ILR implantation.

The study protocol was developed following the principles of the Declaration of Helsinki. Initially, the National Ethics Committee of the Ministry of Healthcare of the Russian Federation approved it, and after that, the center’s local Ethics Committee. All patients signed the written informed consent before enrollment.

The ILR (Reveal XT, Medtronic, USA) was implanted in the left parasternal area at the second intercostal or left axillary region. After ILR implantation and before hospital discharge, we case-by-case, considering the clinical pattern of epileptic seizures in each patient, trained the patients and their relatives on when, how, and how often to use the external activator.

The follow-up duration was up to the end of the life of the ILR battery (approximately 36 months), with scheduled follow-up visits every 3 months. The ILR was removed after the completion of the study or per the patient’s request.

### The ILR activation algorithm

The triggers of ILR autoactivation were cardiac pauses > 3 seconds, bradycardia < 45 beats per min(bpm), ventricular tachycardia (VT) > 150 bpm, rapid ventricular tachycardia > 180 bpm, atrial tachycardia (AT)/atrial fibrillation (AF). The length of stored ECG traces was 30 seconds before and after the automatic activation. For ATs, the programmed duration of the stored ECG recordings preceding the autoactivation was 120 sec.

**Fig. 1:**
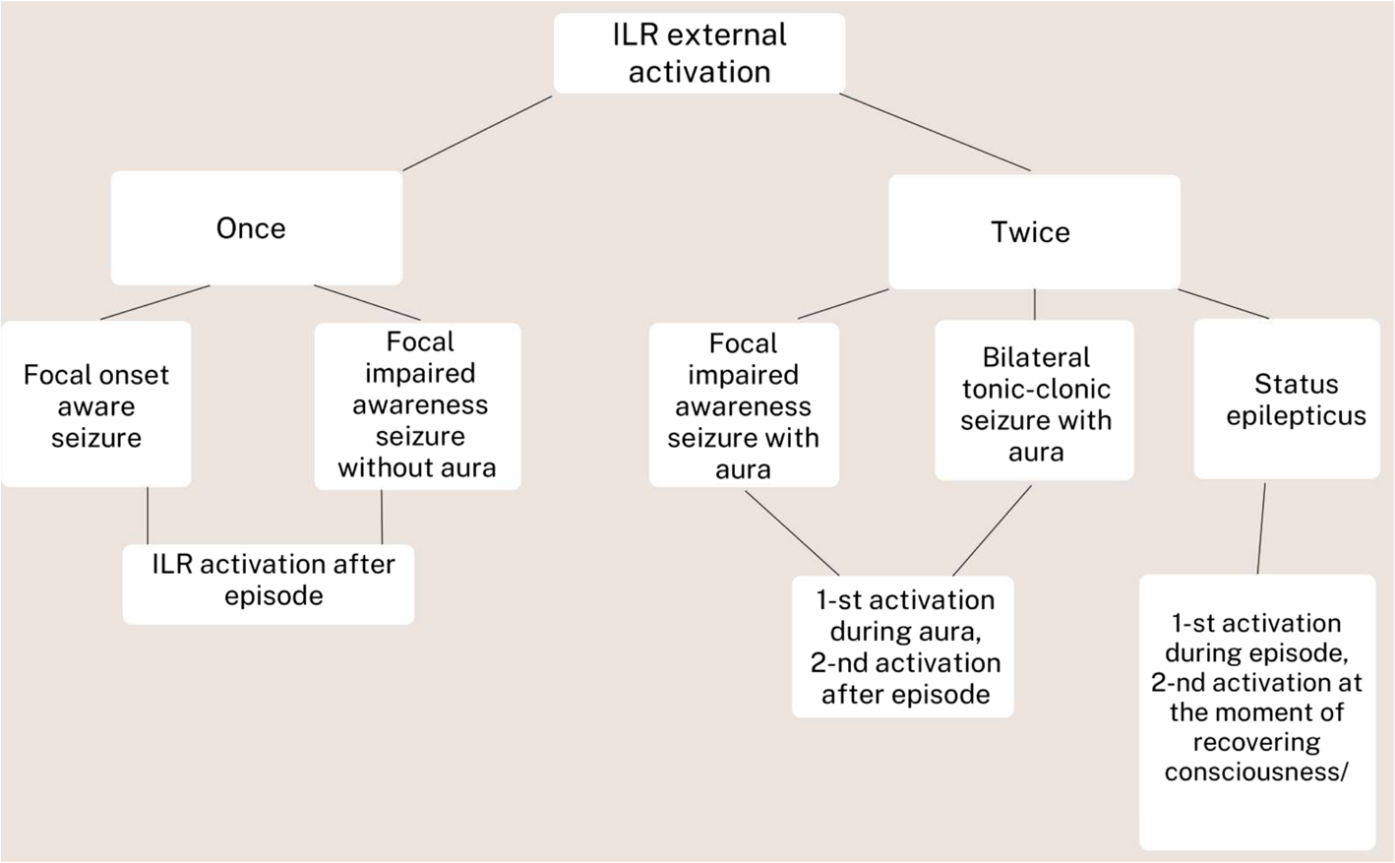
The implantable loop recorder epilepsy type-dependent external activation algorithm

**Fig. 2:**
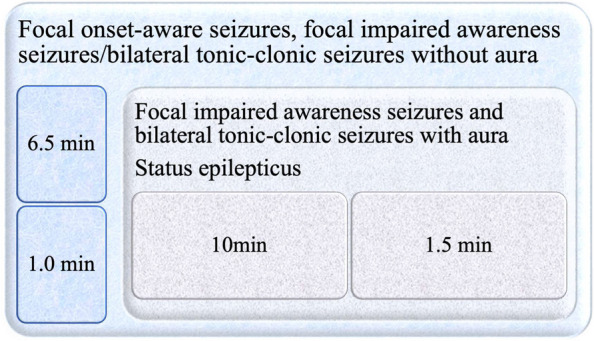
The manual ECG recording setup depends on the epilepsy type

Patients and their relatives were instructed to initiate ECG recording using the manual activator device. Patients with focal onset-aware seizures (FAS) and patients with focal impaired awareness seizures (FIAS)/bilateral tonic-clonic seizures (TCS) without aura were recommended to use the activator once - just after the episode (Fig. 1). In these patients, we set the ILR algorithm to store three ECG traces for up to 6.5 minutes before and 1 minute after the activation (Fig. 2). Patients with FIAS and bilateral TCS with aura were advised to use the activator twice - during the aura and after the episode. The relatives of patients experiencing status epilepticus were instructed to use the activator during the episode and after regaining consciousness. Two ECG strips for up to 10 min before and 1.5 min after the activation were recorded.

The following heart rhythm and rate changes were assessed: cardiac pauses > 3 seconds, bradycardia < 45 bpm, AT/AF, VT > 150 beats/min, rapid ventricular tachycardia > 180 beats/min, sinus tachycardia > 100 bpm, sinus tachycardia > 150 bpm, sinus arrhythmia – variations of PP interval > 10%.

### Statistical analysis

Statistical analysis was performed using SAS software (Version 9·4 software; SAS Institute, Cary, NC, USA). Continuous variables were presented as mean ± standard deviation (SD), median (Me), interquartile range (IQR), and categorical variables – as frequencies. Parametric and nonparametric tests (*t*-test and Kruskal–Wallis test) were used to compare two independent groups. The chi-square and 2-sided Fisher’s tests were used for the categorical variables’ comparison. A two-tailed *p*-value ≤0.05 was regarded as significant.

## Results

The median follow-up duration was 36 [3–36] months. In total, 6494 ECG traces were recorded and analyzed. 4826 of them were auto-triggered events; manual-activated events were 1668. The rate of positive events in the autoactivation group was 19.4% (936 ECG traces). The number of true positive events in the manual-activated group was 1209 (72.5%), statistically higher (19.4% vs. 72.5%, *p* < 0.0001) than in the autoactivation group.

The false-positive events were seizure-induced artifacts, mimicking pseudo-ventricular tachycardia/ventricular fibrillation (VT/VF) episodes, pseudo-AF (sinus arrhythmia mimicking AF), and nocturnal sinus bradycardia.

### The ILR external activation results and epilepsy type

**Fig. 3:**
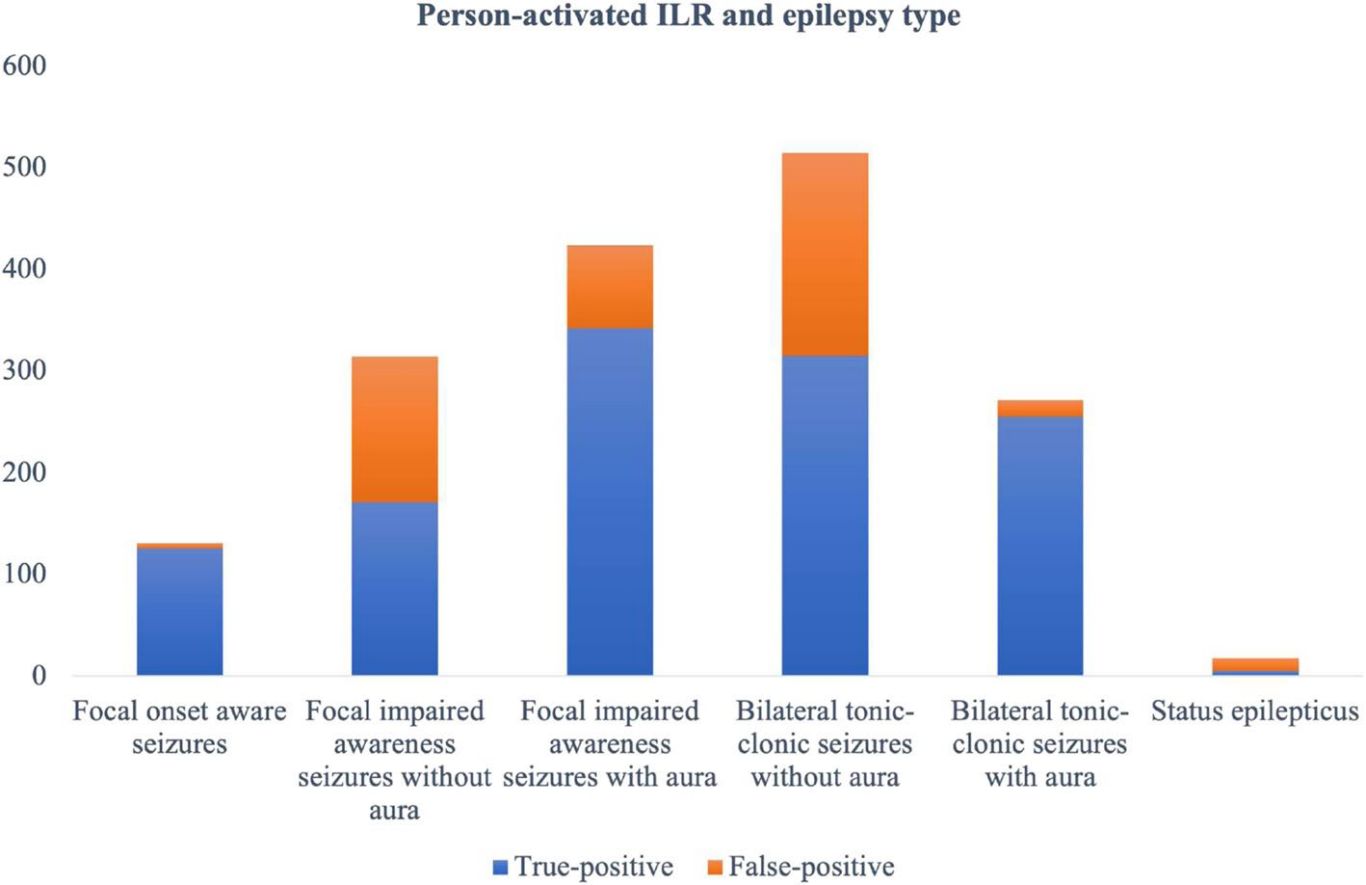
Person-activated ILR-detected true-positive and false-positive events in patients with different types of epilepsy

Figure 3 presents the number of detected true-positive and false-positive events depending on epilepsy type.

Manual-activated false-positive events were observed in approximately one-third of patients with impaired awareness seizures (225 from 738 (30.5%) in patients with FIAS and 216 from 781 (27.7%) in patients with bilateral TCS). The total number of false-positive events in the manual-activated group was 459. The highest rate of false-positive events was detected in patients undergoing epileptic status (13 from 18 to 61.5%); the lowest rate was in patients with FAS (5 from 131 to 3.8%).

**Fig. 4:**
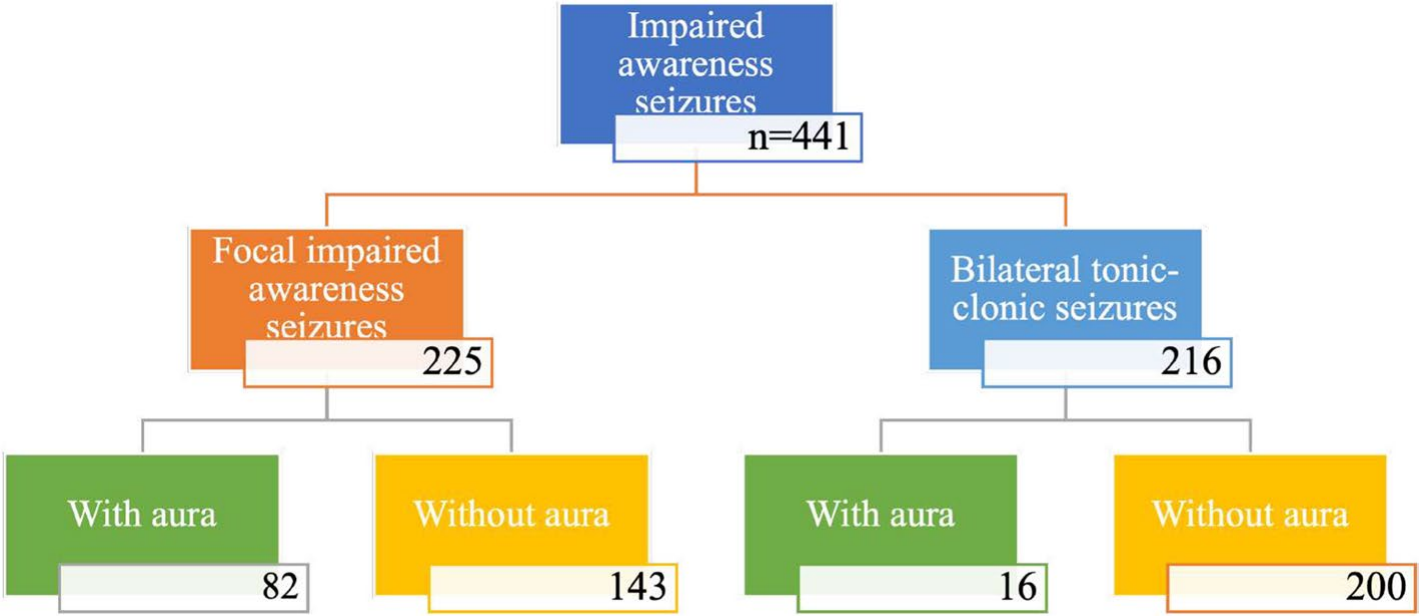
Person-activated ILR false-positive events number in patients with impaired awareness seizures with and without aura

Further subanalysis in patients with impaired awareness seizures revealed a significantly higher rate of false-positive events in patients without aura both in FIAS and bilateral TCS groups (Table 1, Fig. 4).

**Table 1:** False-positive events rate in patients with impaired awareness seizures

Epilepsy type	False-positive events rate	*p*-value
Focal impaired awareness seizures with aura	19.3%	*p* < 0.0001
Focal impaired awareness seizures without aura	45.5%	
Bilateral tonic-clonic seizures with aura	5.9%	*p* < 0.0001
Bilateral tonic-clonic seizures without aura	38.8%	

The comparative analysis between patients suffering from episodes with aura detected a statistically significant higher rate of false-positive events in the group of FIAS (59.8% vs. 38.2%, *p* < 0.0001)

## Discussion

Here we present a real-world experience of the ECG ILR manual activation algorithm in patients with drug-resistant epilepsy. Our algorithm is the first systematized approach to overcome the ECG signal quality compromise [4, 5] in patients with epilepsy, which can mimic cardiac arrhythmias. Based on 6494 ECG traces, our analysis confirmed the high rate of false-positive arrhythmic events in epilepsy patients compromising the ILR memory capacity. In our study, the sensitivity of the built-in ILR autoactivation algorithm was only 19.4%. Such a result would be very far from satisfactory. Contrary to the built-in activation algorithm, the device’s external activation and the correct timing regarding the seizure allowed us to increase the true positive events’ registration rate to 72.5%, thus ensuring our work’s scientific and practical significance.

Initially, the evaluation of ictal cardiac arrhythmias using the ILR was limited by a small sample [6, 7]. In 2004 Rugg-Gunn et al. [6] presented the first results of the continuous evaluation of ictal cardiac arrhythmias via the ILR in 20 patients with focal epilepsy. In this study, in addition to ILR autoactivation, the patients/caregivers were instructed to use the manual activator once after the seizure. The ILR was programmed to store two preceding and subsequent ECG strips of 8 minutes and 2 minutes in length, respectively. The same single-activation approach was used later by M. van der Lende et al. [8]. Although both studies included patients with focal to bilateral tonic-clonic seizures, the authors did not specify the instructions for manual activation depending on epilepsy type. In our research, person-mediated ILR activation once after the seizure was recommended for all patients with FAS and patients with FIAS without aura just after the episode. This approach confirmed its highest sensitivity in patients with FAS. The results for the patients suffering from impaired awareness seizures without aura were satisfactory, with a rate of false-positive events of approximately 40%. The probable cause of such results might be confusion as the relatives/caregivers could not precisely detect whether and when the episode was resolved. The exact cause might be responsible for the highest false-positive events rate in patients undergoing epileptic status.

The rate of false-positive events in patients suffering from seizures with aura was significantly lower in patients with bilateral TCS. In our opinion, a more vivid manifestation of the clinical picture of epileptic seizure in these patients makes it easier to detect the beginning and the end of the epileptic episode.

## Limitations

There are some possible limitations to our study. From the point of view of this paper, the most significant is that we did not use a remote monitoring system for patient management, which could enhance ILR data acquisition and assessment.

## Conclusion

The sensitivity of the built-in ILR autoactivation algorithm in patients with drug-resistant epilepsy is low and seriously compromises the true cardiac arrhythmia detection rate. Our ILR manual activation algorithm is feasible and efficient for improving clinically relevant cardiac arrhythmia detection accuracy.

## Data Availability

The data supporting this study’s findings are available from the corresponding author upon reasonable request.
